# The binding of LARP6 and DNAAF6 in biomolecular condensates influences ciliogenesis of multiciliated cells

**DOI:** 10.1016/j.jbc.2024.107373

**Published:** 2024-05-16

**Authors:** Ryan Earwood, Hiromasa Ninomiya, Hao Wang, Issei S. Shimada, Mia Stroud, Diana Perez, Udval Uuganbayar, Chisato Yamada, Toru Akiyama-Miyoshi, Branko Stefanovic, Yoichi Kato

**Affiliations:** 1 Department of Biomedical Sciences, Florida State University College of Medicine, Tallahassee, Florida, USA; 2 Department of Cell Biology, Nagoya City University, Graduate School of Medical Sciences, Mizuho-ku, Nagoya, Japan; 3 Pathogenic Microbe Laboratory, Research Institute, National Center for Global Health and Medicine, Tokyo, Japan

**Keywords:** cilia, biomolecular condensates, multiciliated cells, DNAAF6, LARP6, *Xenopus*

## Abstract

Motile cilia on the cell surface produce fluid flows in the body and abnormalities in motile cilia cause primary ciliary dyskinesia. Dynein axonemal assembly factor 6 (DNAAF6), a causative gene of primary ciliary dyskinesia, was isolated as an interacting protein with La ribonucleoprotein 6 (LARP6) that regulates ciliogenesis in multiciliated cells (MCCs). In MCCs of *Xenopus* embryos, LARP6 and DNAAF6 were colocalized in biomolecular condensates termed dynein axonemal particles and synergized to control ciliogenesis. Moreover, *tubulin alpha 1c–like* mRNA encoding α-tubulin protein, that is a major component of ciliary axoneme, was identified as a target mRNA regulated by binding LARP6. While DNAAF6 was necessary for high α-tubulin protein expression near the apical side of *Xenopus* MCCs during ciliogenesis, its mutant, which abolishes binding with LARP6, was unable to restore the expression of α-tubulin protein near the apical side of MCCs in *Xenopus* DNAAF6 morphant. These results indicated that the binding of LARP6 and DNAAF6 in dynein axonemal particles regulates highly expressed α-tubulin protein near the apical side of *Xenopus* MCCs during ciliogenesis.

Multiciliated cell (MCC) with motile cilia has been reported in limited organs such as the airways, sinuses, fallopian tubes, and brain ependyma. Motile cilia on these MCCs beat in an oriented manner to generate fluid flows that are important for mucociliary clearance, fertilization, and spinal fluid circulation. Genetic mutations, which disrupt motile cilia function, cause primary ciliary dyskinesia (PCD) which presents as chronic upper and lower respiratory tract infections from childhood, male and female subfertility, visceral inversion, and cardiac defects (1, 2, 3). PCD is an inherited disorder of clinical and genetic heterogeneity, resulting from mutations in over 50 genes mostly involved in the structure and function of motile cilia.

Dynein axonemal assembly factors (DNAAFs) are localized in the cytoplasm and mutations of cytoplasmic DNAAFs lead to a loss of axonemal dyneins, which impairs ciliary motility (4, 5, 6, 7, 8, 9, 10, 11, 12, 13, 14). DNAAF6, a member of DNAAFs, is present within Golgi in human nasal epithelial cells and plays a role in dynein assembly through the interaction with dynein axonemal intermediate chain 2, an intermediate dynein chain of outer dynein arms (11, 15). Dynein axonemal intermediate chain 2 is coassembled with DNAI1 during the first steps of cytoplasmic outer dynein arm preassembly (11, 15, 16, 17). A recent study in the MCCs of *Xenopus* embryos revealed that DNAAF6 is sequestered in G3BP stress granule assembly factor 1 positive biomolecular condensates termed dynein axonemal particles (DynAPs) together with other DNAAFs, chaperons, and subunits of dynein arms (18). Since DNAAFs concentrate with axonemal dyneins and chaperones in DynAPs, DynAPs display the hallmark of biomolecular condensates important for dynein assembly in MCCs (18, 19). The alternative model of axonemal dynein heavy chain (HC) synthesis involving DynAPs is that HC polysomes cross-linked *via* partially synthesized HCs during translation of *HC* mRNAs in DynAPs complete HC synthesis and release fully formed dyneins (20). DynAPs are also enriched in RNAs that have not been identified yet, but not in other stress granule proteins or P-body proteins (18, 21). In addition, cilia- and flagella-associated protein 44 encoded by a human ciliopathy gene and required for cilia motility (22, 23, 24, 25) is an RNA-associated protein localized in DynAPs (21). Since RNA-associated proteins influences the transport and localization of mRNAs, as well as their stability and translation (26, 27, 28, 29), DynAPs may also regulate mRNA metabolism and be requird for the posttranscriptional regulation of gene expression in a variety of physiological and pathological processes.

While we previously reported that a role of RNA-associated protein LARP6, which is a member of the LARP family that carries a La motif (LAM) and a RNA-recognition motif (RRM) (30, 31, 32), regulates the expression of *MCIDAS*, a master factor for the formation of MCCs (33), in MCCs of *Xenopus* embryonic epidermis during ciliogenesis (34), it remains unclear how LARP6 regulates ciliogenesis and what RNAs interact with LARP6 in this process. Here, we extend these observations to elucidate the molecular mechanism underlying ciliogenesis regulated by LARP6. DNAAF6 was isolated as a LARP6-associated protein by yeast two-hybrid screening, and the interaction of LARP6 and DNAAF6 was confirmed in both human cells and *Xenopus* embryos. The functional significance of this interaction has been analyzed and suggests that LARP6 and DNAAF6 colocalized in DynAPs of *Xenopus* MCCs plays an important role to control the highly expressed α-tubulin protein near the apical side of MCCs during ciliogenesis.

## Results

### DNAAF6 is a LARP6-binding protein

**Figure 1:**
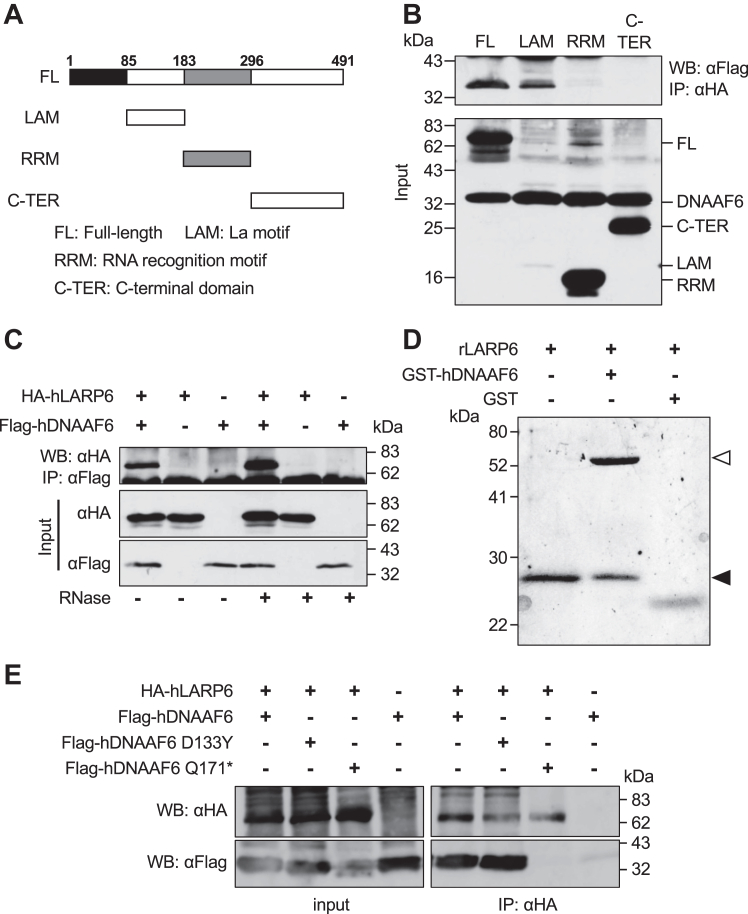
**DNAAF6 interacts with LARP6.***A,* the domain structure of LARP6. The numbers of amino acid are shown on the FL of LARP6. *B,* the La motif is crucial for the interaction between LARP6 and DNAAF6. The tagged LARP6 and DNAAF6 proteins were expressed in mammalian cells. HA-tagged full-length LARP6 (FL), LAM, RRM, and C-TER domain of hLARP6 were immunoprecipitated and Flag-tagged hDNAAF6 analyzed in the precipitate. *Upper panel*: coprecipitated DNAAF6 with LARP6 isoforms; *lower panel:* proteins in input. *C,* LARP6 associates with DNAAF6 in an RNA-independent manner. Flag-tagged hDNAAF6 was immunoprecipitated and HA-tagged FL hLARP6 was analyzed in the precipitate. The protein extract was untreated (lanes 1–3) or treated with RNase (lanes 4–6) before coimmunoprecipitation. *D,* GST-hDNAAF6 pulled down rLARP6. In the *middle lane*, GST-hDNAAF6 indicated by a *white arrowhead* and rLARP6 indicated by a *black arrowhead* were observed. *E,* interaction of the DNAAF6 mutant proteins with LARP6. HA-tagged FL hLARP6 was immunoprecipitated and WT or mutant DNAAF6 proteins were analyzed in the precipitate (lanes 5–8). Lanes 1 to 4 are proteins in the input. The mutant DNAAF6 D133Y (lane 6, *lower panel*) could bind to LARP6, while DNAAF6 Q171∗ could not (lane 7, *lower panel*). C-TER: C-terminal domain; DNAAF, dynein axonemal assembly factor; FL, full length; GST, glutathione-*S*-transferase; GST-hDNAAF6, GST-fused hDNAAF6; HA, hemagglutinin; hDNAAF6, human DNAAF6; hLARP6, human LARP6; LARP6, La ribonucleoprotein 6; RRM, RNA-recognition motif; rLARP6, recombinant human LARP6.

LARP6 has been reported to regulate ciliogenesis in the MCCs of *Xenopus* embryonic epidermis (34). Since it remains unclear how LARP6 regulates ciliogenesis, the associated proteins of LARP6 were searched by yeast two-hybrid screening using the LAM of LARP6, which is an extremely well conserved in various eukaryotic species and able to interact with other proteins (31), as a bait (Fig. 1*A*). This screening identified DNAAF6 as an interacting protein of LARP6, and the interaction between human LARP6 (hLARP6) and human DNAAF6 (hDNAAF6) was confirmed by coimmunoprecipitations from human cell extracts with overexpressed hLARP6 and hDNAAF6 (Fig. 1, *B* and *C*) or purified recombinant human LARP6 (rLARP6) containing only the LAM and RRM (35) and glutathione-*S*-transferase (GST)-fused hDNAAF6 (GST-hDNAAF6) (Fig. 1*D*). In agreement with the result of yeast two-hybrid screening, we found that the LAM of LARP6 is responsible for the interaction between these two proteins (Fig. 1*B*, upper panel) and LARP6 directly interacts with DNAAF6 (Fig. 1*D*, middle lane). Importantly, LARP6 interacted with DNAAF6 in an RNA-independent manner because digestion with RNase did not affect the interaction (Fig. 1*C*).

Mutations of *DNAAF6* have been reported to cause PCD (9, 11, 12, 36, 37). One disease causing mutant of *DNAAF6*, DNAAF6 D133Y, coimmunoprecipitated with LARP6, while the other pathogenic mutant with deletion of 44 amino acids at the C terminus, DNAAF6 Q171∗, did not (Fig. 1*E*, right lower panel). This indicated the importance of DNAAF6 C terminal for the interaction with LARP6.

### *Xenopus* DNAAF6 is required for ciliogenesis

**Figure 2:**
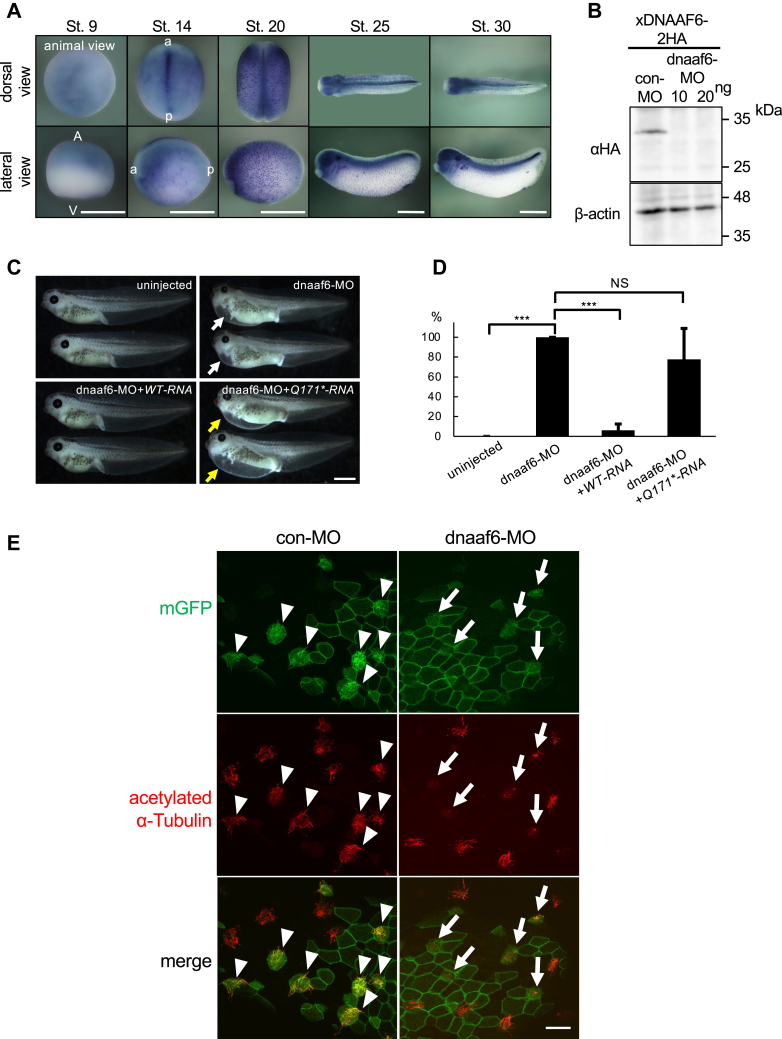
**DNAAF6 is crucial for ciliogenesis in *Xenopus* embryos.***A,* the spatial expression of *xDNAAF6* in *Xenopus* embryos. Developmental stages (St) are indicated on the *top* of each image. A, animal side; V, vegetal side; a, anterior side; p, posterior side. The scale bars represent 1 mm. *B,* efficacy of MO knock down of *Xenopus* DNAAF6. *xDNAAF6-2HA* mRNA encoding HA-tagged *Xenopus* DNAAF6 was coinjected with con-MO (40 ng per embryo) or dnaaf6-MO (10 or 20 ng per embryo), and expression of xDNAAF6-2HA protein from stage 20 embryos was examined by Western blot. Loading control: β-actin. *C, Xenopus* DNAAF6 morphants develop ventral edemas. *Left upper panel:* uninjected embryos. *Right upper panel:* edema indicated by *white arrows* is observed in *Xenopus* DNAAF6 morphants. *Left* and *right lower panels*: formation of edema was rescued by expression of *Flag-xDNAAF6* mRNA (*WT-RNA*) (*left lower panel*), but not by expression of *Flag-hDNAAF6 Q171∗* mRNA (*Q171∗-RNA*) (*right lower panel*: edemas are indicated by *yellow arrows*). The scale bar represents 1 mm. *D,* scoring of edema formation (number of embryos with edema per total number of examined embryos) in *Xenopus* DNAAF6 morphants. Numbers of embryos analyzed: con-MO: 65, dnaaf6-MO: 52, dnaaf6-MO+*WT-RNA*: 53, dnaaf6-MO+*Q171∗-RNA*: 43. ∗∗∗ *p* < 0.001. *E,* dnaaf6-MO inhibited the formation of cilia in *Xenopus* epidermal MCCs. Cilia were stained by an antibody against acetylated α-tubulin (*red*), membrane GFP (mGFP) was used as a tracer of injections. Cilia in control MCCs are indicated by *white arrowheads*, cilia in dnaaf6-MO injected MCCs are indicated by *white arrows*. The scale bar represents 20 μm. Con-MO, control-MO; DNAAF, dynein axonemal assembly factor; HA, hemagglutinin; hDNAAF6, human DNAAF6; MCC, multiciliated cell; MO, morpholino oligo.

Although previous reports demonstrated that loss of LARP6 induces abnormal ciliary morphologies and DNAAF6 is important for axonemal dynein assembly (9, 11, 34), the role of LARP6 and DNAAF6 binding in ciliogenesis remains unknown. To address this question, *Xenopus* was used as an animal model of embryogenesis. The spatial and temporal expression of *DNAAF6* (*xDNAAF6*) in *Xenopus* embryos was first examined by whole mount *in situ* hybridization (Fig. 2*A*). The expression of *xDNAAF6* started in the animal hemisphere at the blastula stage (stage 9) and then high expression was detected in the center of neural plate at the neurula stage (stage 14). The expression of *xDNAAF6* in discrete epidermal cells, started at the neurula stage, was very prominent at stage 20 and was observed until the late tailbud stage (stage 30). As the expression of xDNAAF6 protein was detected only in DynAPs of *Xenopus* epidermal MCCs (18, 19), the cells where *xDNAAF6* mRNAs was detected are very likely the precursors cells of MCC at the neurula stage or MCCs at the tailbud stage. *xDNAAF6* was also expressed strongly in the central nervous system at the tailbud stage.

We demonstrate that the high efficacy of antisense morpholino oligo against *Xenopus DNAAF6* (dnaaf6-MO) to inhibit translation of *xDNAAF6* mRNA (Fig. 2*B*). The expression of *Xenopus* DNAAF6-2HA (xDNAAF6-2HA) protein, which was translated from *xDNAAF6-2HA* mRNA with the sequence of dnaaf6-MO target, was completely abolished in *Xenopus* embryos. When xDNAAF6 was knocked down by dnaaf6-MO, the embryos developed pronounced edema on the ventral side (Fig. 2*C*: edema is indicated by white arrows in right upper panel, Fig. 2*D*). Injection of *xDNAAF6* mRNA (*WT-RNA*), which is not recognized by the dnaaf6-MO, rescued the formation of edema in *Xenopus* DNAAF6 morphants (Fig. 2*C*: left lower panel, Fig. 2*D*), indicating that the *Xenopus* DNAAF6 morphant phenotype is specifically due to depletion of xDNAAF6.

Since the formation of edema suggests ciliary defect in embryos (38), the ciliary morphology on *Xenopus* epidermal MCCs was examined next. Acetylated α-tubulin is a marker of cilia, and we observed that acetylated α-tubulin positive cilia were almost abolished in MCCs of *Xenopus* DNAAF6 morphants (Fig. 2*E*: see MCCs indicated by arrows in right middle panel). These results showed that xDNAAF6 is required for ciliogenesis in *Xenopus* embryos.

### LARP6 is colocalized with DNAAF6 in DynAPs of *Xenopus* epidermal MCCs

**Figure 3:**
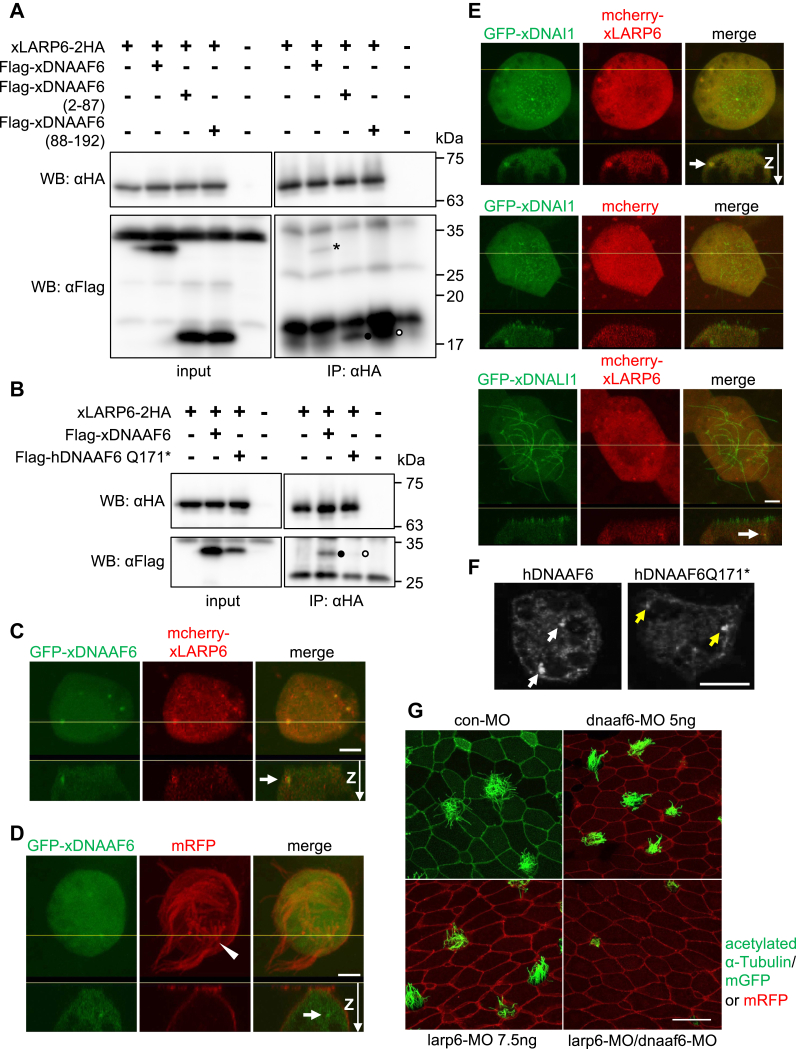
**LARP6 associates and functionally synergizes with DNAAF6 in *Xenopus* embryos.***A,* coimmunoprecipitation of xLARP6-2HA with Flag-xDNAAF6, Flag-xDNAAF6 (2-87) or Flag-xDNAAF6 (88-192) (lane 2, 3 or 4 in the *right lower panel*) using protein extract from stage ten embryos. LARP6 binds the full length of DNAAF6 indicated by a *black asterisk*, DNAAF6 (2-87) indicated by a *black circle* and DNAAF6 (88-192) indicated by a *white circle*. As a note, xDNAAF6 (88-192) includes the protein interacting with HSP90 domain. Expression of xDNAAF6 proteins in the input is shown in the *left lower panel*. Input or immunoprecipitation of xLARP6 is shown in the *right or left upper panel*, respectively. *B,* coimmunoprecipitation of HA-tagged FL xLARP6 with Flag-tagged WT xDNAAF6 indicated by a *black circle* (lane 2 in the *right lower panel*) or human DNAAF6 Q171∗ mutant indicated by a *white circle* (lane 3 in the *right lower panel*). Expression of the proteins from stage 20 embryos in the input is shown in the *left panels* (lanes 1–4). *C*, the colocalization of DNAAF6 and LARP6 in *Xenopus* epidermal MCCs. Fluorescently labeled xDNAAF6 (GFP-xDNAAF6: *green*) and xLARP6 (mcherry-xLARP6: *red*) were expressed in the cytoplasm of MCCs and fluorescence intensity was imaged. The colocalization of GFP-xDNAAF6 and mcherry-xLARP6 was detected as foci. One of these foci is indicated by a *white arrow* in the cytoplasm of MCCs. Each *lower panel* shows the view in x and z plane of the z-stack at the *yellow line* for each *upper panel*. The scale bar represents 5 μm. *D,* the localization of xDNAAF6 and cilia in *Xenopus* epidermal MCCs. GFP-xDNAAF6 (*green*) was detected as foci in the cytoplasm of MCCs with mRFP-positive cilia indicated by a *white arrowhead*. A typical focus is indicated by a *white arrow*. Each *lower panel* shows the view in x and z plane of the z-stack at the *yellow line* for each *upper panel*. The scale bar represents 5 μm. *E,* the colocalization of xDNAI1 or xDNALI1 and xLARP6 in *Xenopus* epidermal MCCs. Fluorescently labeled xDNAI1 (GFP-xDNAI1: *green*) or xDNALI1 (GFP-xDNALI1) and mcherry-xLARP6 were expressed in the cytoplasm of MCCs and fluorescence intensity was imaged. mcherry protein was used as a negative control (a *middle panel* in the *middle row*). The colocalization of GFP-xDNAI1 or GFP-xDNALI1 and mcherry-xLARP6 was detected as foci in the cytoplasm of MCCs. A typical focus is indicated by a *white arrow*. Each *lower panel* shows the view in x and z plane of the z-stack at the *yellow line* for each *upper panel*. The scale bar represents 5 μm. *F,* the localization of human DNAAF6 and human DNAAF6Q171∗ in stage 23 *Xenopus* embryo epidermal MCC. Flag-human DNAAF6 WT (hDNAAF6) or Flag-human DNAAF6Q171∗ (hDNAAF6Q171∗) were expressed in *Xenopus* embryos, and then whole mount immunohistochemistry was performed. Both hDNAAF6 and hDNAAF6Q171∗ were detected as foci indicated by *white arrows* and *yellow arrows* in the cytoplasm of epidermal MCCs, respectively. Confocal images of MCCs at an arbitrary z level. The scale bar represents 10 μm. *G,* LARP6 and DNAAF6 functionally synergize in ciliogenesis of MCCs. mGFP was used as an injection tracer for con-MO (*left upper panel*) and membrane RFP (mRFP) was used as an injection tracer for dnaaf6-MO and/or larp6-MO (all other panels). Cilia were stained by an acetylated α-tubulin antibody (*green*). The scale bar represents 20 μm. DNAAF, dynein axonemal assembly factor; HA, hemagglutinin; hDNAAF6, human DNAAF6; LARP6, La ribonucleoprotein 6; MCC, multiciliated cell; MO, morpholino oligo; RFP, red fluorescence protein; xDNAAF6, *Xenopus* DNAAF6; xDNAI1, *Xenopus* DNAI; xLARP6, *Xenopus* LARP6.

To corroborate the importance of interaction between LARP6 and DNAAF6 in ciliogenesis, this interaction in *Xenopus* embryos was first confirmed by coimmunoprecipitation (Fig. 3*A*). In addition, while both the N-terminal (2-87) and C-terminal (88-192) fragments of xDNAAF6 is necessary for the interaction with *Xenopus* LARP6 (xLARP6), xLARP6 seems to bind the C-terminal fragment of xDNAAF6 more strongly than N-terminal fragment (Fig. 3*A*: an interacting band is indicated by an asterisk, a black circle, or a white circle in the right lower panel, respectively). While WT of human or *Xenopus* DNAAF6 bound xLARP6, the interaction of xLARP6 with hDNAAF6 Q171∗ mutant was weaker than the interaction of xLARP6 with *Xenopus* or human WT DNAAF6 (Fig. 3*B*: an interacting band is indicated by a black or white circle in the right lower panel, Fig. S1*A*: an interacting band is indicated by a black or white circle in the lowest panel). Together with the result that injection of human *DNAAF6Q171∗* mRNA (*Q171∗-RNA*) was unable to rescue the formation of edema in *Xenopus* DNAAF6 morphants (Fig. 2*C*: edema is indicated by yellow arrows in right lower panel), this supports the notion that failure of interaction between LARP6 and DNAAF6 leads to the defects of ciliogenesis and the formation of edema in *Xenopus* DNAAF6 morphants.

The spatial expression of xLARP6 and xDNAAF6 proteins was further confirmed in the MCCs of *Xenopus* embryonic epidermis (Fig. 3, *C* and *D*). As previously published, the expression of xDNAAF6 was detected as foci in the cytosol of MCCs that presumably represent DynAPs (18) (Fig. 3*C*: left panel shows five such foci, Fig. 3*D*). Most sites of xLARP6 accumulation overlapped with the xDNAAF6 foci in the MCC (Fig. 3*C*: one such site is indicated by an arrow in right panel). *Xenopus* DNAI1 (xDNAI1) and DNALI1 (xDNALI1) have been reported to be expressed in DynAPs in *Xenopus* epidermal MCCs (18, 19) (Fig. S2*A*). xLARP6 foci also overlapped with xDNAI1 and xDNALI1 in *Xenopus* epidermal MCCs (Fig. 3*E*: one such site is indicated by an arrow in each right panel), showing that xLARP6 colocalizes with xDNAAF6 and dynein subunits in DynAPs of *Xenopus* epidermal MCCs. In addition, hDNAAF6 and DNAAFQ∗171 were also detected as foci in the cytosol of *Xenopus* epidermal MCCs (Fig. 3*F*).

The functional synergy between LARP6 and DNAAF6 in ciliogenesis was also tested by simultaneously knocking down both proteins. We first determined the highest amount of larp6-MO or dnaaf6-MO, which alone is insufficient to induce the abnormal ciliary morphologies in the MCCs (Fig. 3*G*: right upper and left lower panels). When these submorphant amounts of larp6-MO and dnaaf6-MO were coinjected, the formation of cilia was inhibited in the MCCs (Fig. 3*G*: right lower panel). If the binding frequency of xLARP6 and xDNAAF6 within a certain period is important for ciliogenesis and assuming that it is possible to reuse the reduced protein by submorphant amounts of MO, reducing both may result in a lower binding frequency than just one. This may be why xLARP6 and xDNAAF6 functionally synergized each other in MCC ciliogenesis. Taken together, the results shown in this figure suggested that the binding of xLARP6 and xDNAAF6 plays a significant role in DynAPs of MCCs leading to productive ciliogenesis.

### LARP6 rapidly fluxes through DynAPs

**Figure 4:**
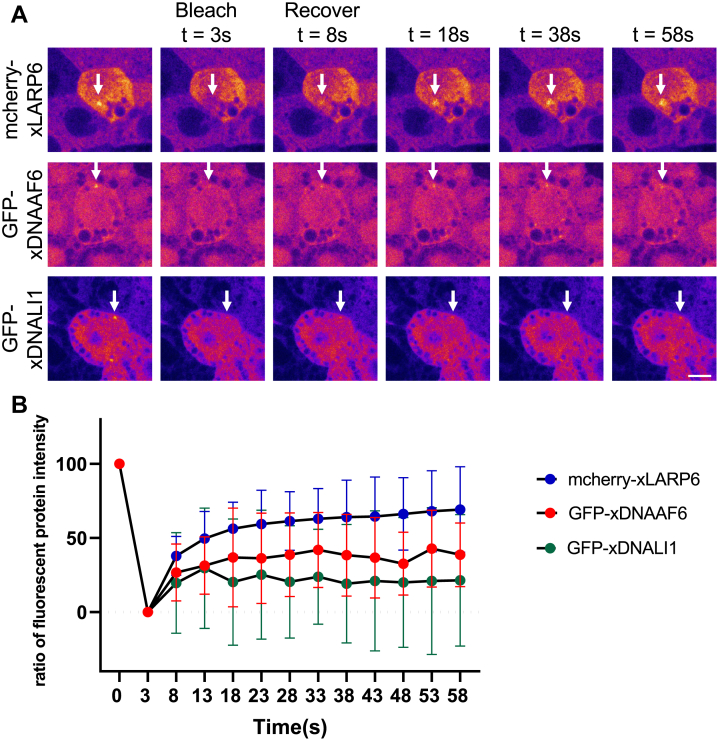
**LARP6 showed the high mobility into DynAPs after bleaching.***A,* time lapse images of mcherry-xLARP6, GFP-xDNAAF6, and GFP-xDANLI1 fluorescence recovery after photobleaching. “s” indicates seconds. Each *white arrow* indicates a fucus of fluorescently labeled protein. The scale bar represents 10 μm. *B,* fluorescence recovery after photobleaching kinetics of mcherry-xLARP6 (n = 48), GFP-xDNAAF6 (n = 23), and GFP-xDNALI1 (n = 17). “n” indicates the number of counted DynAPs. The *y*-axis shows the ratio of fluorescent protein intensity compared with the intensity in the same area before bleaching. DynAP, dynein axonemal particle; LARP6, La ribonucleoprotein 6; xDNAI1, *Xenopus* DNAI1; xDNAAF6, *Xenopus* DNAAF6; xLARP6, *Xenopus* LARP6.

Previous study with fluorescence recovery after photobleaching (FRAP) demonstrated that DNAAFs fluxed rapidly through DynAPs of *Xenopus* epidermal MCCs, while dynein subunits were stably retained (18). Since xLARP6 binds xDNAAF6 and localizes in DynAPs as showed above, we expected xLARP6 to flux rapidly through these organelles, similar to DNAAFs (18). We performed FRAP experiments with two controls such as xDNAAF6 that rapidly fluxes through DynAPs and xDNALI1 that is stably retained (18). xLARP6 (mcherry-xLARP6 in upper panels of Fig. 4*A*) showed rapid FRAP kinetics after bleaching of entire DynAPs, suggesting rapid exchange of xLARP6 between DynAPs and the cytoplasm (Fig. 4, *A* and *B*). As xLARP6 showed the high mobility after bleaching like DNAAFs which do not localize to axonemes and play as cytoplasmic assembly factors of cilia, xLARP6 and xDNAAF6 may function together to generate motile cilia in *Xenopus* epidermal MCCs.

We also tested if xDNAAF6 is required for the localization of xLARP6 within DynAPs. The colocalization of xDNAI1 and xLARP6 was examined in MCCs of *Xenopus* DNAAF6 morphants. The colocalization of xDNAI1 and xLARP6 in DynAPs of DNAAF6 depleted MCCs was not changed compared with control MCCs (Fig. S3, *A* and *B*), indicating that the accumulation of xLARP6 within DynAPs is independent of xDNAAF6.

### The interaction of LARP6 and DNAAF6 is crucial for fluid flow generated by motile cilia

**Figure 5:**
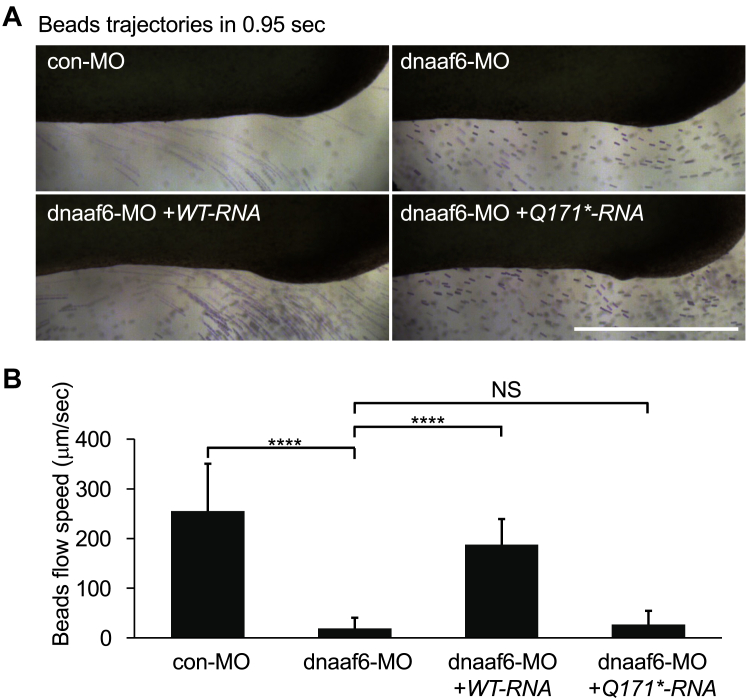
**DNAAF6 is required for fluid flow generated by motile cilia on *Xenopus* epidermal MCCs.***A,* the flow generated by cilia in *Xenopus* epidermal MCCs. Trajectories of beads (*purple*) were imaged within 0.95 s in MMR buffer of control embryos (*left upper panel*) and *Xenopus* DNAAF6 morphants (*right upper panel*). The trajectories after coinjection of *WT-RNA* (*left lower panel*) or *Q171∗-RN*A (*right lower panel*) with dnaaf6-MO is shown in the *bottom panels*. The scale bar represents 1 mm. *B,* quantification of the speed of beads flow. Number of embryos examined; con-MO: 16, dnaaf6-MO: 15, dnaaf6-MO +*WT-RNA*: 16, dnaaf6-MO +*Q171∗-RNA*: 16. ∗∗∗∗ <0.0001. C-TER, C-terminal domain; Con-MO, control-MO; DNAAF, dynein axonemal assembly factor; MCC, multiciliated cell; MMR, Marc's modified Ringer's solution; MO, morpholino oligo; RRM, RNA-recognition motif.

To confirm the importance of interaction between xLARP6 and xDNAAF6 in the function of motile cilia on the MCCs, the flow generated by the MCC cilia was examined. Colored beads were added into the embryonic buffer and the flow rates of beads were measured (Fig. 5, Movie S1, *A*–*D*). The flow generated by motile cilia of *Xenopus* DNAAF6 morphants was dramatically reduced, but it was almost completely restored by coinjection of *WT-RNA* (Fig. 5*A*: right upper panel and left lower panel, Fig. 5*B*). On the other hand, coinjection of *Q171∗-RNA* restored only the negligible flow generated by motile cilia of *Xenopus* DNAAF6 morphant (Fig. 5*A*: right lower panel, Fig. 5*B*). As the interaction of xLARP6 and hDNAAF6 Q171∗ is weaker than the interaction of xLARP6 with WT DNAAF6 (Fig. 3*B*, Fig. S1*A*), this result supported the hypothesis that the interaction of xLARP6 and xDNAAF6 impacted on the formation of motile cilia, as well as the mobility of cilia in *Xenopus* epidermal MCCs.

### The binding of LARP6 and DNAAF6 regulates the expression of α-tubulin proteins in *Xenopus* epidermal MCCs

**Figure 6:**
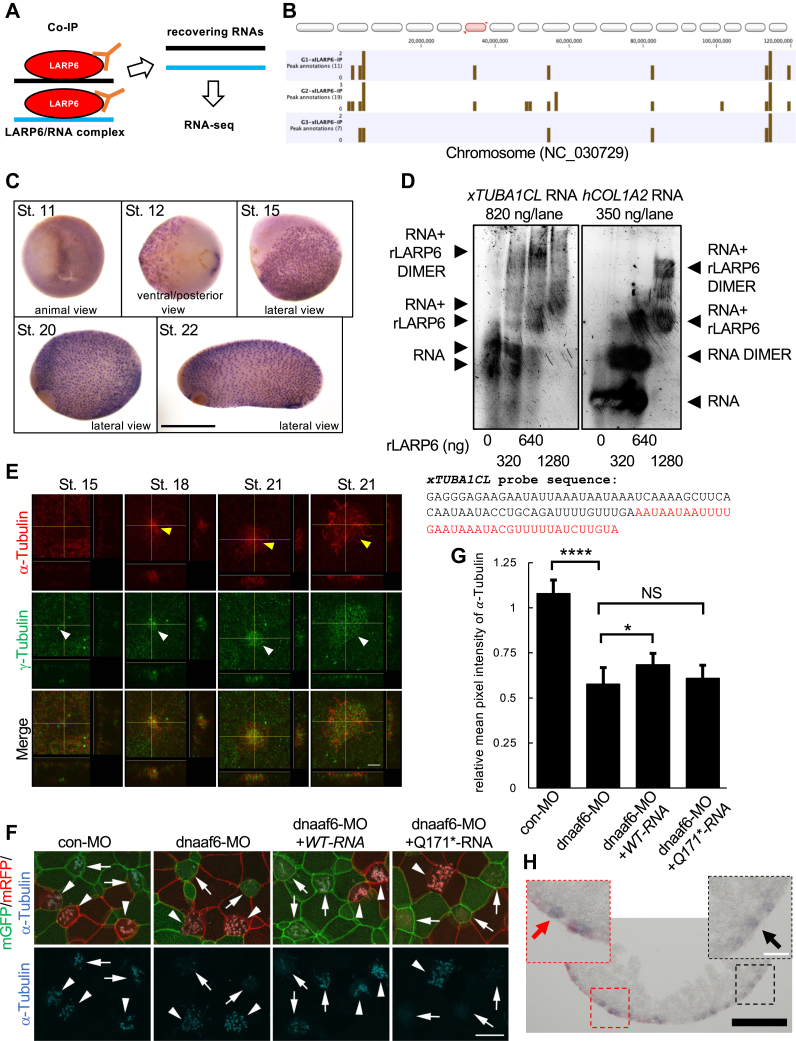
**LARP6 binds *xTUBA1CL* mRNA and DNAAF6 is important for the expression of α-tubulin proteins in *Xenopus* epidermal MCCs.***A,* the strategy of screening for *Xenopus* LARP6–interacting RNAs in *Xenopus* embryos is shown. Three independent coimmunoprecipitations were performed and pull-down RNAs were analyzed by RNA-seq. *B,* peak annotations in the *red-highlighted* part of chromosome (NC_030729) are shown. *Brown bars* represent peak annotations. G1-xlLARP6-IP, G2-xlLARP6-IP, and G3-xlLARP6-IP were sample names of coimmunoprecipitation experiments. *Numbers in parentheses* show the number of peak annotations in a *red-highlighted* chromosome. *C,* the spatial expression of *xTUBA1CL* mRNA in *Xenopus* embryos. WISH was performed with *xTUBA1CL* RNA probe. As a note, *xTUBA1CL* RNA probe likely recognizes mRNAs encoding α-tubulin. St. indicates developmental stages. The scale bar represents 1 mm. *D,* recombinant human LARP6 protein (rLARP6) binds *xTUBA1CL* mRNA. *Left panel: xTUBA1CL* probe (RNA) with the sequence shown was incubated with increasing amounts of rLARP6, and RNA/Protein complexes were resolved on a native acrylamide gel. *Right panel:* the same experiment with human *Collagen type I alpha 2 chain* (*hCOL1A2*) RNA probe was shown as a positive control. Mobility of free RNA and that of RNA/rLARP6 monomer and dimer complexes are shown (*arrowheads*). The *red-highlighted* part in *xTUBA1CL* probe sequence is the precipitated sequence by xLARP6. *E,* α-tubulin protein is highly expressed in *Xenopus* epidermal MCCs at the onset of ciliogenesis. The signals of α-tubulin protein (*red*) at different embryonic stages are indicated by *yellow arrowheads* (*upper panels*). The signals of γ-tubulin (*green*) are indicated by *white arrowheads* and represent MCCs or precursor MCCs (*middle panels*). The merged image is shown in the *bottom panels*. Two panels from the right show different cells at the same stage. The view of x and z plane of the z-stack at the *yellow line* or the view of y and z plane of the z-stack at the *yellow line* in *left upper large square* was shown under or on the *right of left upper large square*, respectively. The scale bar represents 5 μm. *F,* the expression of α-tubulin protein was abolished in stage 23 *Xenopus* DNAAF6 morphants. *mRFP* mRNA was injected alone into one cell of two-cell stage embryos, and *mGFP* mRNA was coinjected with con-MO, dnaaf6-MO, dnaaf6-MO/*WT-RNA,* or dnaaf6-MO/*Q171∗-RNA* into the other side of cell at four-cell stage embryos. Arrows represent MCCs, into which *mGFP* mRNA and con-MO, dnaaf6-MO, dnaaf6-MO/*WT-RNA,* or dnaaf6-MO/*171Q∗-RNA* were delivered, while *arrowheads* indicate control MCCs into which only *mRFP* mRNA was delivered. The expression of α-tubulin protein (*cyan*) is observed in cilia. The scale bar represents 20 μm. *G,* the relative mean pixel intensity of α-tubulin protein staining. The relative mean pixel intensity is mean pixel intensity of GFP-positive MCCs relative to mean pixel intensity of adjacent RFP-positive MCCs. ∗: *p* < 0.05, ∗∗∗∗: *p* < 0.0001. *H,* the expression of *xTUBA1CL* mRNA in stage 20 *Xenopus* DNAAF6 morphants. SISH was performed with *xTUBA1CL* RNA probe. Transverse section at the ventral side of embryo is shown. As dnaaf6-MO was coinjected with β-galactosidase mRNA, dnaaf6-MO–injected side is Red-Gal positive side of embryo (*left side*). The expression level of *xTUBA1CL* mRNA (*purple*) is not much different between DNAAF6 knockdown cells and control cells. The area surrounded by a *red or black dotted square* is enlarged. A *red arrow* or a *block arrow* indicates a typical signal of *xTUBA1CL* mRNA, respectively. The *black and white scale bars* represent 200 and 50 μm, respectively. Con-MO, control-MO; MCC, multiciliated cell; MO, morpholino oligo; RFP, red fluorescence protein; SISH, section *in situ* hybridization; xLARP6, *Xenopus* LARP6; xTUBA1CL, *Xenopus* tubulin alpha 1c like.

The localization of xDNAAF6 is limited to DynAPs of *Xenopus* epidermal MCCs and DynAPs are rich in RNAs (18, 21). Therefore, the function of interaction between xLARP6 and xDNAAF6 in *Xenopus* epidermal MCCs may be to regulate the metabolism or posttranscriptional regulation of critical mRNAs, so the target RNAs that can be recognized by xLARP6 were searched by the combination of coimmunoprecipitation and RNA-seq (Fig. 6*A*). *Type I collagen* mRNAs have been identified as the high affinity targets of LARP6 (35), but recent individual-nucleotide resolution UV cross-linking and immunoprecipitation experiments suggested that LARP6 may bind other mRNAs, as well (39). Three independent coimmunoprecipitations with xLARP6-2HA–overexpressed and uninjected *Xenopus* embryos were performed and recovered RNAs were sequenced. After sequencing data were compared between xLARP6-2HA–overexpressed and control uninjected samples, the RNAs whose peak shape scores were more than 1.5 in all three independent experiments were considered as binding candidates of xLARP6 (Data S1). In addition, an example of peak annotations in three independent experiments was shown in Fig. 6*B*. For further analysis, *Xenopus tubulin alpha 1c–like* (*xTUBA1CL*) mRNA encoding α-tubulin protein was selected from the group of precipitated RNAs, because α-tubulin is a major component of ciliary axoneme (40). Also, the expression of *xTUBA1CL* mRNA was detected like the expression of *xDNAAF6* mRNA in the epidermis of *Xenopus* embryos (Fig. 6*C*). To confirm that LARP6 can bind *xTUBA1CL* mRNA *in vitro*, the gel mobility shift assay was performed. Short radiolabeled RNA with the sequence of *xTUBA1CL* mRNA (nt 1468-1585 of XM_018238729.2) was incubated with rLARP6 (35), and the RNA/protein complex were resolved on a native gel. *xTUBA1CL* RNA probe bound to rLARP6, forming monomeric and dimeric complexes (Fig. 6*D*: left panel). As a control experiment, the interaction of rLARP6 and human *collagen type I alpha 2 chain* mRNA or its mutant is shown (Fig. 6*D*: right panel, Fig. S4*A*). This known RNA target of LARP6 WT but not mutant also formed monomeric and dimeric complexes. These experiments verified that *xTUBA1CL* mRNA can be recognized by LARP6 *in vitro* and it can be regulated by LARP6 *in vivo*. To identify the motifs of target RNAs which xLARP6 binds, we performed multiple em for motif elicitation (MEME) analysis with our data (DRA015067) and other group’s data (E-MTAB-9636) (39). The first sequences from our data in Fig. S5*A* and the second sequences from other group in Fig. S5*B* similarly included multiple uridines, indicating the similarity of LARP6-binding motifs in different experimental systems.

Since the high expression of α-tubulin protein was detected near the apical side of *Xenopus* epidermal MCCs at the onset of ciliogenesis (around stage 18) (Fig. 6*E*: top panel, Fig. S6*A*), the expression of α-tubulin protein was examined in DNAAF6-depleted MCCs of *Xenopus* embryonic epidermis. The expression of α-tubulin protein near the apical side of MCC precursors was strikingly reduced in *Xenopus* DNAAF6 morphant at stage 20 and 23 (Figs. 6, *F*, *G* and S6*B*). This reduction was restored by coinjection of *WT-RN*A but not *Q171∗-RNA* (Fig. 6, *F* and *G*). On the other hand, the expression of *xTUBA1CL* mRNA is already detected in epidermis at stage 12 and the level of its expression remains high through at least stage 22 (Fig. 6*C*). The expression level of *xTUBA1CL* mRNA was not much different between DNAAF6 knockdown cells and control cells in stage 20 *Xenopus* DNAAF6 morphant epidermis (Fig. 6*H*). To examine the quantity of *xTUBA1CL* mRNA and α-tubulin protein in epidermal cells of *Xenopus* embryos including MCCs, *Xenopus* ectodermal explants that differentiate into epidermis (41) were used. The expression levels of x*TUBA1CL* mRNA and α-tubulin protein were not changed between control-MO (con-MO)-injected explants and dnaaf6-MO–injected explants at stage 25 (Fig. S6, *C*, *D* and *E*). These results indicated the binding of xLARP6 and xDNAAF6 in DynAPs may be important for temporal and spatial expression level of α-tubulin protein within *Xenopus* epidermal MCC precursors or MCCs, but not changes in the expression levels of *xTUBA1CL* mRNA and α-tubulin protein, during ciliogenesis.

## Discussion

In this work, we have shown that DNAAF6 is physically associated with LARP6 in human cultured cells and *Xenopus* embryos. Edema and dysfunction of cilia on MCCs was observed in *Xenopus* DNAAF6 morphants and these defects were rescued by expression of WT *DNAAF6* mRNA but not *DNAAF6Q171∗* mRNA that cannot interact with DNAAF6. xLARP6 is colocalized with xDNAAF6 in biomolecular condensates DynAPs of MCCs and showed the high mobility to these organelles like xDNAAF6. Furthermore, xLARP6 functionally synergized with xDNAAF6 during ciliogenesis in *Xenopus* embryonic epidermis. *xTUBA1CL* mRNA encoding α-tubulin protein was identified as one of mRNAs recognized by xLARP6. Highly expressed α-tubulin protein near the apical side of MCCs at the beginning of ciliogenesis was abolished in *Xenopus* DNAAF6 morphants and DNAAF6Q171∗ could not restore its expression. These results suggest that the binding of xLARP6 and xDNAAF6 in DynAPs is crucial for the high expression of α-tubulin protein to respond to the beginning of ciliogenesis in *Xenopus* MCCs. Here, we reported another role of xLARP6 through the interaction with xDNAAF6 during ciliogenesis in MCCs of *Xenopus* embryonic epidermis, apart from the role that controlled the expression of a master transcriptional factor MCIDAS for ciliogenesis (34).

Biomolecular condensates have crucial roles in maintaining homeostasis, stress responses, development, and disease (42, 43, 44). In particular, the functions of RNA-rich condensates have been reported to be involved in long-range transport and on-demand translation of RNA (45, 46, 47) as well as storage of RNA (48). Since DynAPs, which are important for the assembly of dynein arms in motile cilia (19), are enriched in RNAs (21), they may have other functions related to the post-transcriptional or translational regulation of RNA in addition to those already known. While the punctate expression of *xTUBA1CL* mRNA, which is a xLARP6 binding target, was first detected in epidermis at the late gastrula stage and the level of its expression remains high through at least tailbud stage, the high expression of α-tubulin protein appears near the apical side of MCCs at late neurula stage. A time difference between the transcription and translation of *xTUBA1CL* gene was observed, suggesting that xLARP6 likely controls the timing of translation from *xTUBA1CL* mRNA in *Xenopus* epidermal MCCs. On the other hand, highly expressed α-tubulin protein near the apical side of MCCs during ciliogenesis disappeared in *Xenopus* DNAAF6 morphants, but the expression levels of *xTUBA1CL* mRNA and α-tubulin protein were not reduced by the depletion of DNAAF6 in *Xenopus* ectodermal explants which are identical to epidermal cells. This indicated that xDNAAF6 seems to regulate the spatial expression of α-tubulin protein within *Xenopus* epidermal MCCs. Moreover, xLARP6 is colocalized with xDNAAF6, which functionally synergize with xLARP6 in ciliogenesis of MCCs, in DynAPs of MCCs during ciliogenesis, indicating that binding of xLARP6 and xDNAAF6 in DynAPs influences ciliogenesis of MCCs. Putting these findings together, the binding of xLARP6 and xDNAAF6 in DynAPs is possibly involved in the temporal and spatial regulation of translation from *xTUBA1CL* mRNA.

An important practical implication of our work is that DNAAF6 Q171∗, a mutant of DNAAF6 found in a PCD patient, could not interact with LARP6 and not restore defects of *Xenopus* DNAAF6 morphants, such as morphology and motility of cilia in *Xenopus* epidermal MCCs. This suggests that the basis for pathogenesis underlying some forms of this disorder may be the inability of patients to form a functional interaction of LARP6 and DNAAF6. On the other hand, the motile cilia in nasal mucosa of a PCD patient with complete loss of DNAAF6 due to deletion of exon 1 to 5 and 5′-flanking noncoding region showed defects of dynein arms and a decreased number of peripheral microtubules (12). This indicates that complete loss of DNAAF6 protein mainly impacts the formation of dynein arms. As compared to *Xenopus* DNAAF6 morphants, the defects of ciliary morphology in *Xenopus* epidermal MCCs were more severe than the defect in the PCD patient mentioned above. This difference may be explained by genetic compensation as described in the zebrafish animal model. In zebrafish, acute knockdown of CEP290 by MO caused severe cilia-related phenotypes, but the deficiency of *CEP290* induced by CRISPR/Cas9 gene editing was mild and restricted to photoreceptor defects (49). While the upregulation of genes encoding the cilia-associated small GTPases were observed in the milder CRISPR/Cas9 phenotypes, the upregulation of these small GTPases was not sufficient in zebrafish CEP290 morphants, indicating that gene compensation occurs and contributes to genotype–phenotype variations between the mutants and morphants. By a similar mechanism, acute knockdown of DNAAF6 in *Xenopus* embryos induced more severe morphology of motile cilia in epidermal MCCs as compared with the motile cilia in a PCD patient in whom DNAAF6 protein was genetically lost. Even considering this caveat, we presented evidence that dnaaf6 is involved in the initiation of rapid α-tubulin protein production in response to the onset of ciliogenesis in MCCs.

Finally, our findings here suggest an undiscovered role of xLARP6 and xDNAAF6 binding localized in RNA-rich biomolecular condensates DynAPs during ciliogenesis of *Xenopus* MCCs. Further studies are necessary to understand the mechanistic aspects of how the binding of xLARP6 and xDNAAF6 regulates the temporal and spatial expression of α-tubulin protein in *Xenopus* MCCs during ciliogenesis.

## Experimental procedures

### *Xenopus laevis* embryo manipulations

Eggs were artificially fertilized by using testis homogenates and cultivated in 0.1 × Marc's modified Ringer's solution (MMR) (50). Embryos were staged according to Nieuwkoop and Faber (51). The studies using *Xenopu*s were approved by review boards of Florida State University (FSU) and Nagoya City University.

### Screening of associated proteins with LARP6

Yeast two-hybrid screening was done by cloning hLARP6 fragment, aa 73-303, into pGBKT7 vector and used it as a bait to screen Clontech Mouse Embryonic Fibroblast Matchmaker cDNA library (Clontech, ML-4009AH) containing 3.5 × 10^6^ independent clones for the His auxotrophy and LacZ activation according to the manufacturers protocol (Clontech, PT3247-1). A clone expressing full size *DNAAF6* was obtained.

### DNA constructs

HA-hLARP6 (pCDNA3-HA-hLARP6) and xLARP6-2HA (pCS2+-xLARP6-2HA) were previously used (34, 35). For Flag-hDNAAF6 (pCS2+-3Flag-hDNAAF6), hDNAAF6 was subcloned into BglII-XhoI sites of pCS2+-3Flag vector. For hemagglutinin (HA)-hLARP6 LAM (pCDNA3-HA-hLARP6 LAM), HA-hLARP6 RRM (pCDNA3-HA-hLARP6 RRM) or HA-hLARP6 C-TER (pCDNA3-HA-hLARP6 C-TER), hLARP6 LAM, and hLARP6 RRM or hLARP6 C-TER was subcloned into BamHI-XhoI sites or KpnI-XhoI of 2HA-pCDNA3 vector, respectively. For GST-hDNAAF6 (pGEX6P-2-hDNAAF6), hDNAAF6 was subcloned into BamHI-XhoI sites of pGEX6P-2. For pCR2.1-xDNAAF6, *Xenopus* DNAAF6 was subcloned into pCR2.1-TOPO vector using TA cloning procedure (Thermo Fisher Scientific, 451641). For Flag-xDNAAF6 (pCS2+-3Flag-xDNAAF6), *Xenopus* DNAAF6 was subcloned into EcoRI-XbaI sites of pCS2+-3Flag vector. For xDNAAF6-2HA (pCS2+-xDNAAF6-2HA), *Xenopus* DNAAF6 was subcloned into EcoRI-StuI sites of pCS2+-2HA vector. For mchery-xLARP6 (pCS105-mcherry-xLARP6), xLARP6 was cloned into BamHI-AgelI sites of pCS105-mcherry vector. For GFP-xDNAAF6 (pCS2+-eGFP-xDNAAF6), *Xenopus* DNAAF6 was subcloned into BglII-NotI sites of pCS2+-eGFP vector. For GFP-xDNALI1 (pCS2+-eGFP-xDNALI1), *Xenopus* DNALI1 was cloned into EcoRI-XhoI sites of CS2+-eGFP vector. For GFP-xDNAI1 (pCS2+-eGFP-xDNAI1), xDNAI1 was cloned into EcoRI-XhoI sites of CS2+-eGFP vector. For pBS-KS-xTBUA1CL, xTUBA1CL fragment was cut out from pTA2-xTBUA1CL by BamHI and SalI, and then was cloned into BamHI-SalI sites of pBS-SK(II) vector. Flag-hDNAAF6 D133Y (pCS2+-3Flag-hDNAAF6 D133Y) and Flag-hDNAAF6 Q171∗ (pCS2+-3Flag-hDNAAF6 Q171∗) were made by site directed mutagenesis of the Flag-hDNAAF6 clone using QuikChange mutagenesis kit (Agilent Technologies, #200523-5). For Flag-xDNAAF6 (2-87) (pCS2+-3Flag-xDNAAF6 (2-87)), *Xenopus* DNAAF6 (2-87) was cloned into BglII-XhoI sites of pCS2+-3Flag vector. For Flag-xDNAAF6 (88-192) (pCS2+-3Flag-xDNAAF6 (88-192)), *Xenopus* DNAAF6 (88-192) was cloned into EcoRI-SnaBI sites of pCS2+-3Flag vector. Primers used for subcloning were shown in Table 1.

**Table 1:** Primer sequences

Plasmid name	Forward	Reverse
pCS2+3Flag-hDNAAF6	gcaagatctgggaatctgaaaatatggatt	cgactcgagtcagaagaaattagcaata
pCDNA3-HA-hLARP6 LAM	cgcagatctggaggtgagaacgagcgtga	cggactcgagttagctggggaggttctcgtt
pCDNA3-HA-hLARP6 RRM	gacggtaccatgctcctggtctatgatctctacttg	atgctcgagttatgcatagtccgggacgtcata gggatagcccgcatagtcaggaacatcgtatgggta
pCDNA3-HA-hLARP6 C-TER	cgcggatccaaaaagaaacctgccaaagag	gcgctcgagttatagacaggcggtgctcct
pGEX6P-2-hDNAAF6	aaaagatctatggaatctgaaaatatgaag	aaactcgagtcagaagaaattagcaatatc
pCR2.1-xDNAAF6	tccctgctcaaccatcctac	gcatgatggagcacacaaac
pCS2+-3Flag-xDNAAF6	aagaattcaatggaactggcactgggtga	aatctagatcaaaggaagttgatgaaat
pCS2+-xDNAAF6-2HA	aaagaattccatagtttgaaaatggaactggcactgggtg	aaaaggccttaaggaagttgatgaaatctaattctcg
pCS105-mcherry-xLARP6	SP6 primer in pCS2+ vector	aaaaccggtaaacactgaacgggc
pCS2+-eGFP-xDNAAF6	aaaagatctgaactggcactgg	M13 reverse primer in pCS2+
pCS2+-eGFP-xDNALI1	aaacaattgcatgattcccccggctgattc	aaagtcgacttcttgggtgcaataattccttc
pCS2+-eGFP-xDNAI1	aaacaattgcatgccgactaaacagcgac	aaagtcgacaaattctgctctgtgtgtctg
pTA2-xTUBA1CL	gagggagaagaatattaaat	tacaagataaaaacgtattt
pCS2+-3Flag-xDNAAF6 (2-87)	SP6 primer in pCS2+	aaactcgagctactgctctctcagatccag
pCS2+-3Flag-xDNAAF6 (88-192)	aaagaattcaccagagtatgaggtattatttaag	T3 primer in pCS2+

C-TER, C terminal; DNAAF, dynein axonemal assembly factor; HA, hemagglutinin; hDNAAF6, human DNAAF6; hLARP6, human LARP6; LAM, La motif; RRM, RNA-recognition motif; xDNAAF6-2HA, Xenopus DNAAF6-2HA; xDNAI1, Xenopus DNAI1; xLARP6, Xenopus LARP6; xTUBA1CL, Xenopus tubulin alpha 1c like.

### Coimmunoprecipitation and immunoblotting

For coimmunoprecipitation with cultured cells, HEK293T cells were grown in Dulbecco’s modified Eagle’s medium with 10% of Fetal Plus serum for up to ten passages. HEK293T cells were transfected with 1μg of the indicated expression vectors per 35 mm dish by 293TransIT reagent (Mirus Bio, MIR2704) and harvested 2 to 3 days after transfection. Cell extracts were prepared in lysis buffer (10 mM KCl, 1.5 mM MgCl_2_, 10 mM Tris–HCl (pH 7.5), 0.5% NP-40, protease inhibitors). After centrifugation, the cleared lysate was incubated with 1 μg of antibody for 3 h at 4 °C. Thirty microliters of equilibrated protein A/G-agarose Plus beads (Santa Cruz Biotechnology, sc-2003) were added, and incubation continued for an additional 3 h.

For coimmunoprecipitation with *Xenopus* embryos, embryos were homogenized in 0.5% Triton X lysate buffer (20 mM Tris–HCl [pH 8.0], 5 mM MgCl_2_, 1 mM EDTA, 50 mM KCl, 0.5% Triton X-100, 10% glycerol, and 1 mM DTT, protease inhibitors), and embryonic protein extracts were used for immunoprecipitation. The embryonic protein extracts were incubated with 1 μg of antibody at room temperature for 1 h.

HA (Roche, 11 583 816 001 or Santa Cruz Biotechnology, sc-7392), Flag (Sigma-Aldrich, F3165), α-tubulin (Sigma-Aldrich, T9026), GAPDH (Novus Biologicals, NB300-322), and β-actin (Santa Cruz Biotechnology, sc-69879) antibodies were used for immunoprecipitation or immunoblotting. Protein extracts for immunoblotting were generated from animal caps using 0.5% Triton X lysate buffer. Gel images were obtained by scanning the films or Amersham Imager 680 (GE Healthcare). The intensity of protein bands in immunoblotting images from three independent experiments was measured by ImageJ software (version 1.53t, http://imagej.net/ij/), and the average intensity of protein bands was calculated.

### *In vitro* protein–protein interaction

GST-hDNAAF6 or GST alone was purified on Glutathione Agarose (Sigma-Aldrich, G4510). The purified rLARP6 containing only the LAM and RRM (Cai *et al.*, 2010) was added to the beads containing either GST-hDNAAF6 or GST. After washing, the bound proteins were eluted with free glutathione to release GST-hDNAAF6 or GST and stained with Bio-Safe Coomassie Stain (Bio-Rad, 1610786).

### Whole mount *in situ* hybridization

Embryos were fixed with 3-(N-morpholino)propanesulfonic acid/EGTA/magnesium sulfate/formaldehyde (MEMFA) (0.1 M MOPS, 2 mM EGTA [pH 8.0], 1 mM MgSO_4_, and 3.7% formaldehyde) for 1 h. Whole mount *in situ* hybridization was performed essentially as described previously (52) by using digoxigenin (Sigma-Aldrich, D9026)-labeled antisense RNA probes and BM purple (Sigma-Aldrich, 11442074001) for the chromogenic reaction. pCR2.1-xDNAAF6 or pBS-KS-xTUBA1CL was linearized by Acc65I or BamHI for generating a template and an RNA probe for the detection of *xDNAAF6* or *xTUBA1CL* mRNA was synthesized by *in vitro* transcription with T7 or T3 RNA polymerase (Thermo Fisher Scientific, AM2085 or EP0101).

### Microinjection of synthetic mRNAs and MOs

Capped synthetic mRNAs were generated by *in vitro* transcription with SP6 RNA polymerase, using the mMessage mMachine kit (Thermo Fisher Scientific, AM1340). For microinjections, embryos were injected with 5 to 10 nl of the specified amount of mRNA in 3% Ficoll in 0.1 × MMR and cultured in 0.1 × MMR until the desired stage. The site of injection was determined based on the cell fate map of *Xenopus* embryos (53).

*Membrane RFP* mRNA as well as *membrane GFP* mRNA for immunohistochemistry were injected as a lineage tracer. Antisense MO and standard control MO were purchased from Gene Tools. Sequences of MO used in this work are followed.

Con-MO: CCTCTTACCTCAGTTACAATTTATA,

larp6-MO: TCTCCTCGGGCTCCTCCATGTCACT (34)

dnaaf6-MO: CTCACCCAGTGCCAGTTCCATTTTC.

### Whole mount fluorescence immunohistochemistry

Published procedures were used for staining (54) with minor modifications. Embryos were fixed in MEMFA either at room temperature for 2.5 h or at 4 °C overnight for acetylated-α-tubulin (Sigma-Aldrich, T6793) and α-tubulin (Sigma-Aldrich, T9026) and γ-tubulin (abcam, ab11321) antibodies. For Flag (Sigma-Aldrich, F3165) antibody, embryos were fixed in Dent’s fixative (methanol-dimethyl sulfoxide) at −20 °C overnight. Fixed embryos were washed and stored in PBS at 4 °C. After rinsing in 0.1% Triton X-100 in PBS (PBT), embryos were incubated with 10% goat serum in PBT at room temperature for at least 1 h. Samples were incubated with mouse acetylated-α-tubulin antibody (1:500), α-tubulin antibody (1:20,000), γ-tubulin antibody (1:500), and Flag antibody (1:500) at 4 °C overnight. Primary antibodies were recognized with Alexa Flour 594 goat anti-mouse IgG antibody (1:500, Thermo Fisher Scientific, A110050), Cy2 donkey anti-mouse IgG antibody (1:500, Jackson ImmunoResearch, 715-225-150), Alexa Flour 488 goat anti-rabbit IgG antibody (1:500, Thermo Fisher Scientific, A11008), and Alexa Fluor 647 goat anti-mouse IgG antibody (1:500, Thermo Fisher Scientific, A21236), respectively. Antibodies were diluted in 10% goat serum in PBT. Images were taken by confocal microscopy (Zeiss LSM 880: Germany and Olympus FV3000: Japan).

### FRAP

*Xenopus* embryos were imaged with an Olympus FV3000 using a *UPlanXApo* 100x/1.45na Oil Objective. A region of interest was defined for full bleach experiments. Region of interests were bleached using 30% laser power of both 488 nm and 561 nm lasers for 1 s and a 10 μs/pixel dwell time. Fluorescence recovery was recorded every 5 s up to 1 min. The intensity of fluorescent signals was analyzed using FV 3000 software (https://www.olympus-lifescience.com/en/laser-scanning/fv3000/super-resolution-software-module/). The plots were generated using Prism 9.5.1 software (GraphPad Software, https://www.graphpad.com/).

### Beads flow assay

Beads flow assay was performed as previously reported (55, 56). Stage 26 embryo was held by cray mold, and 10 μm latex beads (Polysciences, 17136), stained with Sudan black and diluted in PBS at 0.025%, was poured above head part of the embryo. Immediately after the pouring, pictures were taken consecutively for 5 s in 15 to 20 frames under a dissecting microscope equipped with a digital camera. Positional change of the beads within 0 to 200 μm away from ventral midline of the embryo was measured with ImageJ software (Fiji). In time lapse movie (avi file), dots of the same bead in different times were connected with straight lines (line tool), length of lines were measured, and total length of the lines were summed. Flow velocities of beads were calculated by the total length of lines/total time. Afterward, average flow velocities of beads were calculated and further averaged in each embryo. We measured 4 to 6 beads/embryo of 15 to 16 embryos from four batches for each condition.

### Screening of LARP6-associated RNAs

To identify LARP6-associated RNAs in *Xenopus* embryos, xLARP6-2HA was overexpressed in stage 22 embryos and embryos were used in the immunoprecipitation experiments, followed by detection of RNA using RNA chromatin immunoprecipitation (ChIP)-IT Kit (Active Motif, 53024). Uninjected stage 22 embryos were used as a negative control for the immunoprecipitation experiments. The immunoprecipitation was performed as published procedure with some modifications (57), and RNA elution and reversal of cross-links were performed according to manufacturer's instruction. Fifty *Xenopus* embryos per experimental sample were fixed in 1% formaldehyde in PBS for 1 h to cross-link. Cross-linked embryos were lysed by 0.5% Triton X lysate buffer with RNase inhibitor, and the samples were sonicated using the following conditions: 3 × 20 s, 20% output, QSONICA Q55 sonicator. After DNase I treatment at 22 °C for 10 min, sheared samples were immunoprecipitated with HA antibody (Roche, Switzerland, 11 583 816 001) at 4 °C overnight. Immunoprecipitated samples were washed with wash buffer described in previous publication (57). To elute RNAs and reverse cross-link, washed samples were incubated with RNA-ChIP elution buffer with RNase inhibitor in RNA ChIP-IT Kit at room temperature for 15 min, and then supernatant was treated with 5 M NaCl and Proteinase K at 42 °C for 1 h and subsequently at 65 °C for 1.5 h. After reversal of cross-link, RNAs were purified by ReliaPrep RNA Clean-Up and Concentration System (Promega, Z1071). Complementary DNA (cDNA) for next generation sequencer were generated from recovered RNAs by PrimeScript Double Strand cDNA Synthesis Kit (Takara, 6111A) and purified by MinElute PCR Purification Kit (QIAGEN, 28004).

The cDNA libraries were prepared using SMARTer Stranded RNA-Seq Kit (Takara, 102319) and Nextera XT DNA Sample Preparation kits (Illumina, FC-131-1024) according to the manufacturer's instructions. For multiplexing, the dual index system of the kit was employed. The resulting cDNA library products were sequenced using the MiSeq (Illumina) with a 602-cycle reagent kit to obtain 301 × 2 paired end reads. The obtained raw reads were analyzed by CLC genomics workbench 20.0.4, a commercial software (https://digitalinsights.qiagen.com/products-overview/discovery-insights-portfolio/analysis-and-visualization/qiagen-clc-genomics-workbench/).

### Gel mobility shift assay

The RNA probes were prepared by *in vitro* transcription (HiScribe T7 High Yield RNA Synthesis Kit, New England BioLabs, E2040S). The sequence of the probes is shown in Fig. 5*B* and in the previous publication (35). Eight hundred twenty nanograms of *xTUBA1CL* RNA probe and 350 ng of human *collagen type I alpha 2 chain* RNA probe were incubated with increasing amounts of recombinant human LARP6 (rLARP6) containing only the LAM and RRM (35) in 10 mM Tris pH 7.5, 100 mM KCl and 5 mM MgCl_2_ at room temperature for 10 min. The RNA/protein complexes were resolved on a 6% native gel and visualized by staining of RNA with SYBR Gold dye. The images of gels were taken by Bio-Rad ChemiDoc Imaging System.

### MEME analysis

The consensus RNA sequences which LARP6 binds were analyzed using the MEME analysis (https://meme-suite.org/meme/tools/meme), which discovers motifs that an approximate sequence pattern in a group of related sequences.

### Section *in situ* hybridization

*Xenopus* embryos were fixed with MEMFA for 1 h. Activity of β-galactosidase, coinjected with MO, was detected using Red-Gal substrate (Sigma-Aldrich, RES1364C) just after fixation. After sectioning samples, section *in situ* hybridization was performed essentially as described previously (52, 58) by using digoxigenin-labeled antisense RNA probes and BM purple for the chromogenic reaction.

### quantitative PCR (qPCR) analysis

RNA was isolated from animal caps using ISOGEN II (NIPPON GENE, Japan, 311-07361) and cDNA was synthesized by ReverTra Ace qPCR RT Master Mix with gDNA Remover (TOYOBO, FSQ-301). qPCR analysis was performed with FastStart Universal Master (Roche, 04913914001) using a QuantStudio 12K Flex System (Thermo Fisher Scientific). Primers for *xTUBA1CL* are followed;

F: tatgtaggggaagggatggag

R: ctgcaggtattattgtgaagcttttga

### Statistics

Statistical analyses were performed using one-way ANOVA, followed by Tukey’s multiple comparison test. All statistical analyses were performed using GraphPad Prism Software (version 9.5.1, GraphPad Software). *p* values less than 0.05 indicate significant differences between groups.

## Data availability

The datasets and computer code produced in this study are available in the following databases:

RNA-seq data: DDBJ Sequence Read Archive, DRA015067 (https://www.ddbj.nig.ac.jp/dra/index.html).

## Supporting information

This article contains Supporting information.

## Conflict of interests

The authors declare that they have no conflicts of interest with the contents of this article.

## Supporting information

Data S1

Movie S1A

Movie S1B

Movie S1C

Movie S1D
