# Supplementary material for: The binding of LARP6 and DNAAF6 in biomolecular condensates influences ciliogenesis of multiciliated cells
Source: J Biol Chem. 2024 May 16;300(6):107373. doi: 10.1016/j.jbc.2024.107373 (PMC11208920; doi:10.1016/j.jbc.2024.107373)
Supplement: Supporting Figures [file mmc6.pdf]

Supporting information

Figures

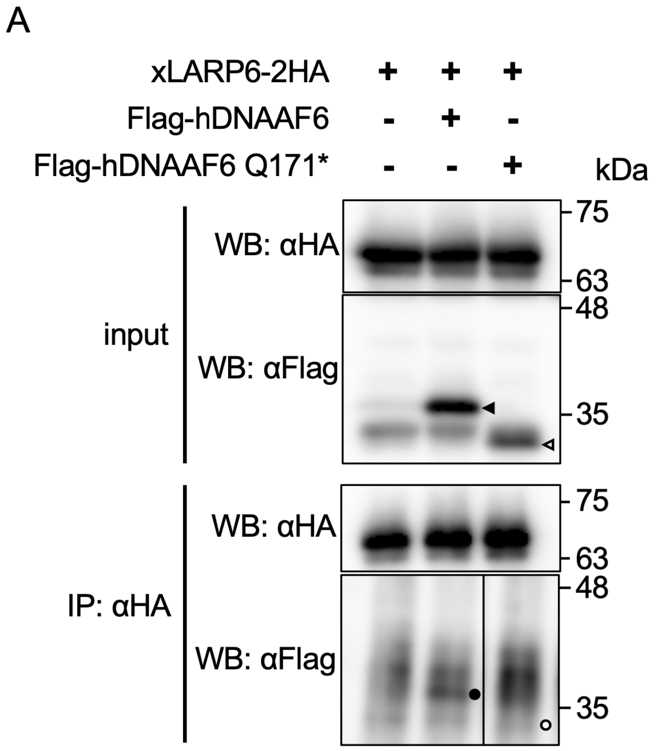

Figure S-1. ***Xenopus*** **LARP6 interacts with human DNAAF6 Q171* mutant more weakly than wild type of human DNAAF6**

B. Co-immunoprecipitation of HA-tagged xLARP6 with Flag-tagged hDNAAF6 indicated by a black circle (lane 2 in the lowest panel) or with hDNAAF6 Q171* mutant indicated by a white circle (lane 3 in the lowest panel). Expression of the proteins from stage 20 embryos in the input is shown in two upper panels (lanes 1-3). Expression of hDNAAF6 or hDNAAF6 Q171* mutant in the input is indicated by a black arrowhead or a white arrowhead, respectively (lane 2 or 3 in the second upper panel).

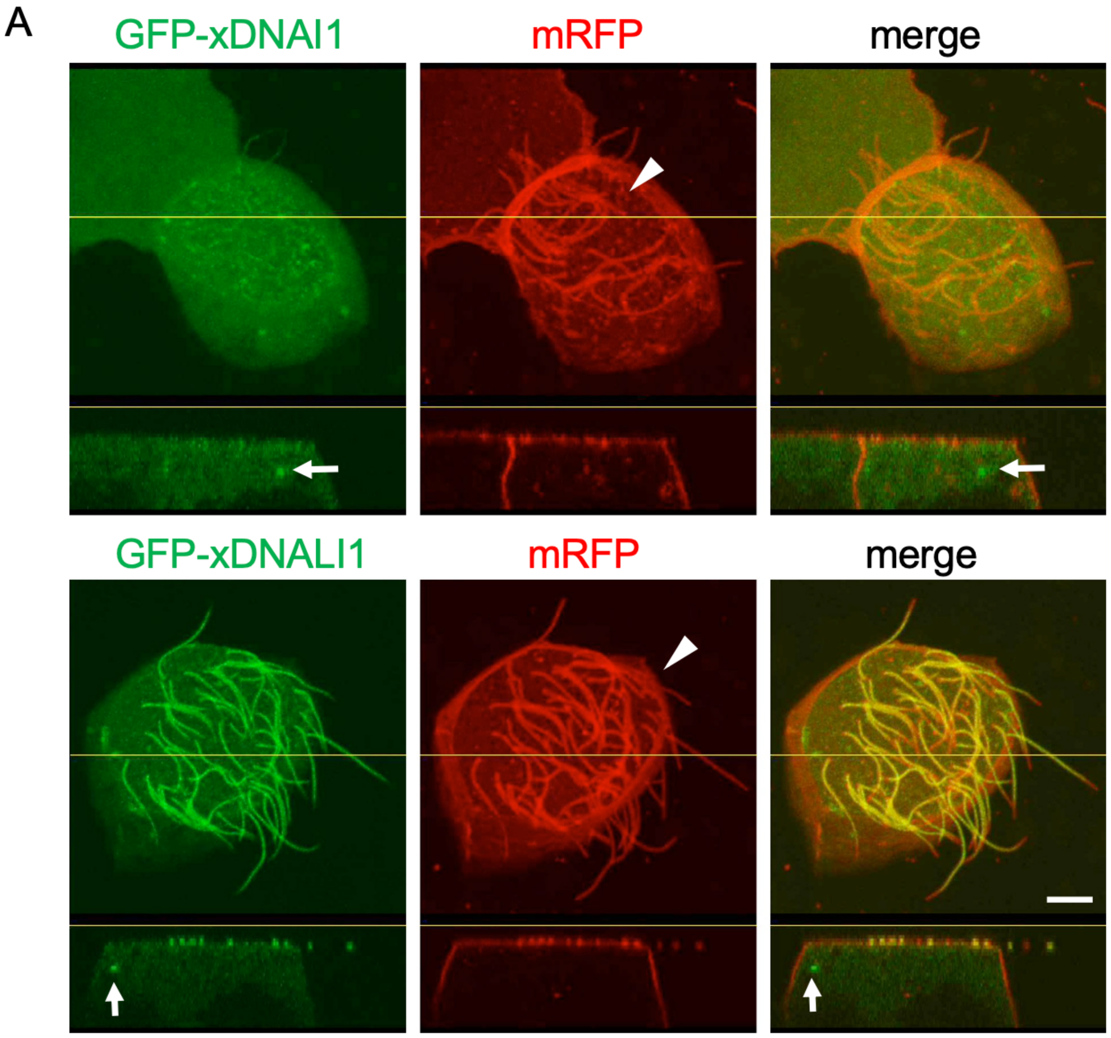

Figure S-2. xDNAI1 and xDNALI1 localizes inside DynAPs of *Xenopus* epidermal MCCs

A. Fluorescently labeled DNAI1 (GFP-xDNAI1: green) and DNALI1 (GFP-xDNALI1) were detected as foci in the cytoplasm of MCCs. A typical focus is indicated by a white arrow. Cilia was fluorescently labeled by membrane RFP (mRFP: red) and indicated by a white arrowhead. Fluorescence intensity was imaged. Each lower panel shows the view in x and z plane of the z-stack at the yellow line for each upper panel. The scale bar represents 5μm.

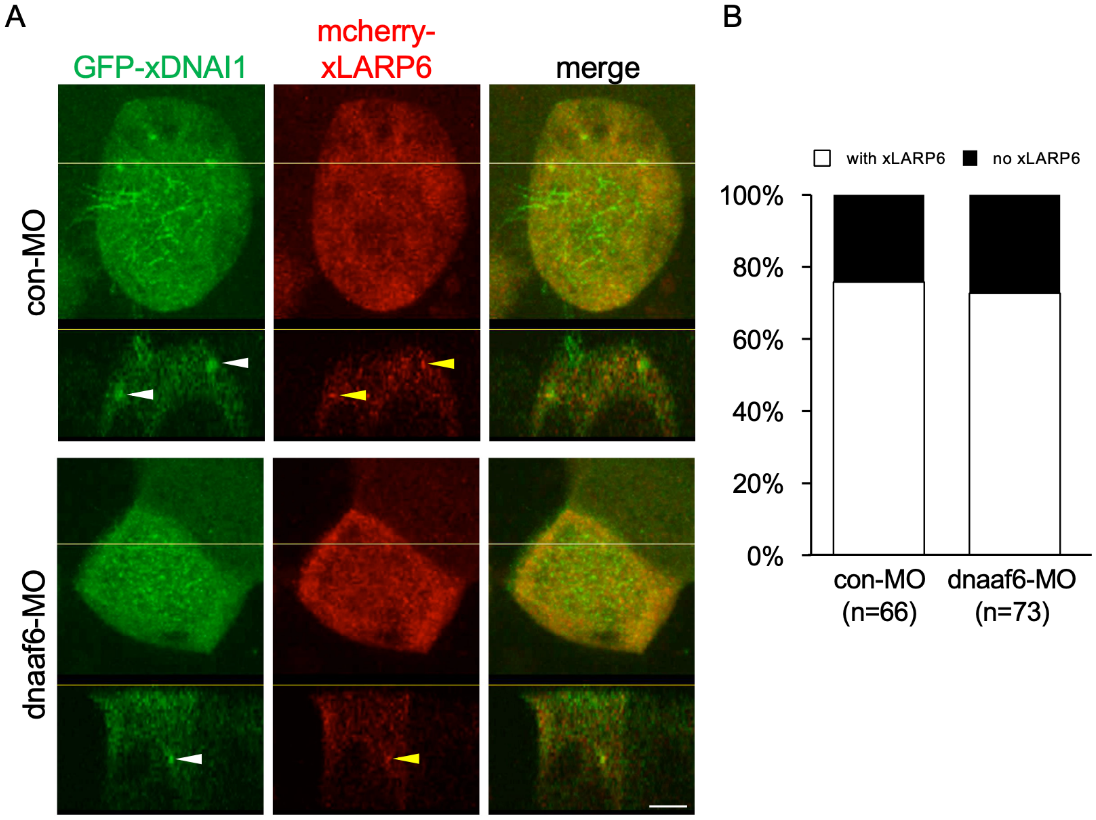

Figure S-3. xDNAAF6 is not necessary for the localization of xLARP6 within DynAPs

A. Fluorescently labeled DNAI1 (GFP-xDNAI1: green) and LARP6 (mcherry-xLARP6: red) were detected as foci in the cytoplasm of MCCs. Typical foci of xDNAI1 or xLARP6 are indicated by white arrowheads or yellow arrowheads, respectively. Fluorescence intensity was imaged. Each lower panel shows the view in x and z plane of the z-stack at the yellow line for each upper panel. The scale bar represents 5μm. B. Scoring of xDNAIl positive DynAPs with xLARP6. n: number of examined xDNAI1 positive DynAPs.

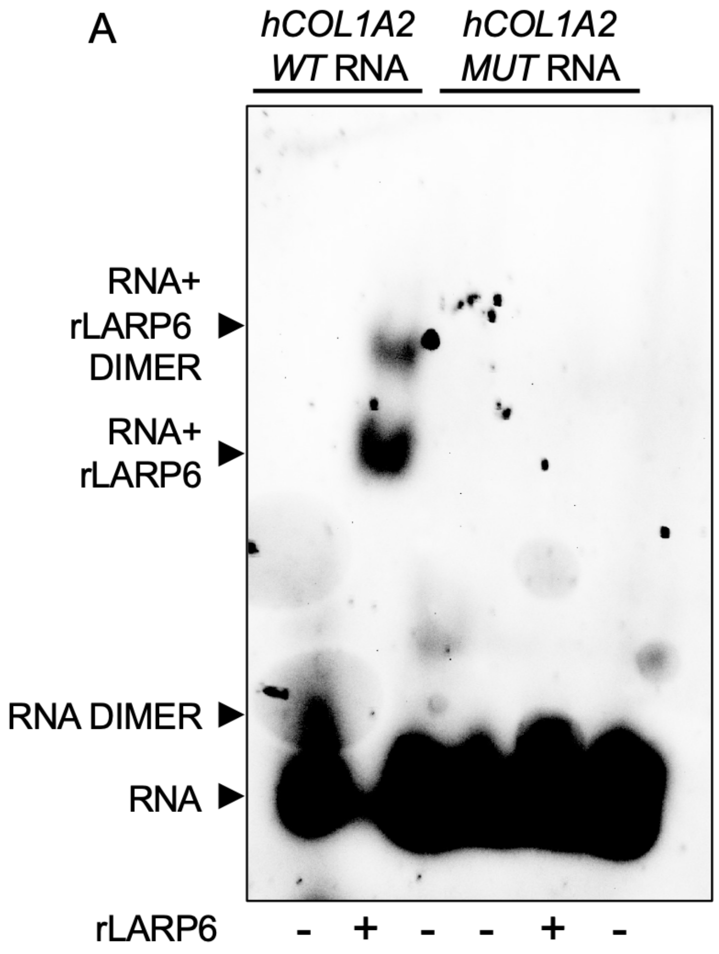

Figure S-4. Mutant of *hCOL1A2* RNA did not interact with rLARP6

A. Wild type of *hCOL1A2* RNA probe (*hCOL1A2* WT RNA) or mutant of *hCOL1A2* RNA probe (*hCOL1A2* MUT RNA) was incubated with rLARP6, and RNA/Protein complexes were resolved on a native acrylamide gel. Mobility of free RNA and that of RNA/rLARP6 monomer and dimer complexes are indicated by arrowheads. The sequence of *hCOL1A2* WT RNA or *hCOL1A2* MUT RNA is followed;

hCOL1A2 WT RNA:

CCACAAAGAGUCUACAUGUCUAGGGUCUAGACAUGUUCAGCUUUGUGG

hCOL1A2 MUT RNA:

CCACAAAGUGACUACAUGUCUAGGGUCUAGACAUGUUAUUCUUUGUGG

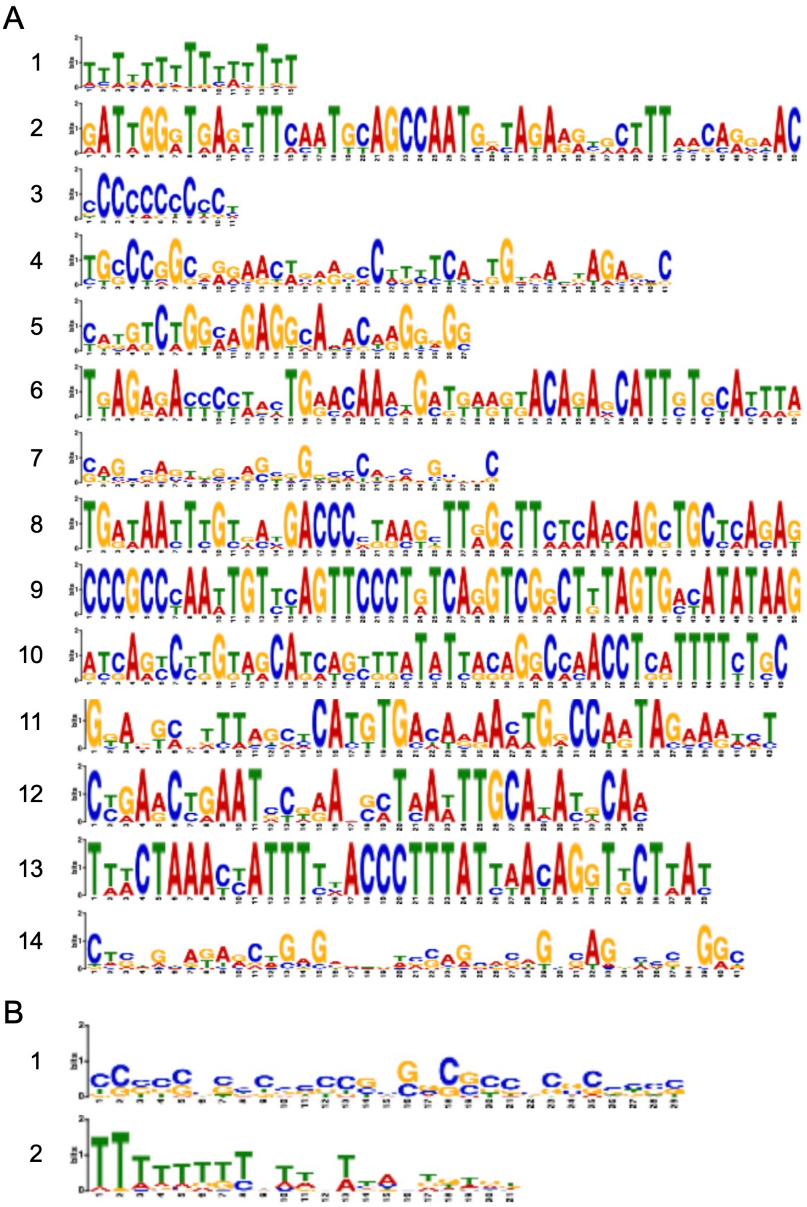

Figure S-5. MEME analysis for LARP6-binding motifs

A. Results of MEME analysis with RNAs isolated from *Xenopus* embryos co-immunoprecipitated by xLARP6. B. Results of MEME analysis with RNAs isolated from MDA-MB231 cells co-immunoprecipitated by LARP6. The positions marked thymidine are the positions of uridine in RNA sequences.

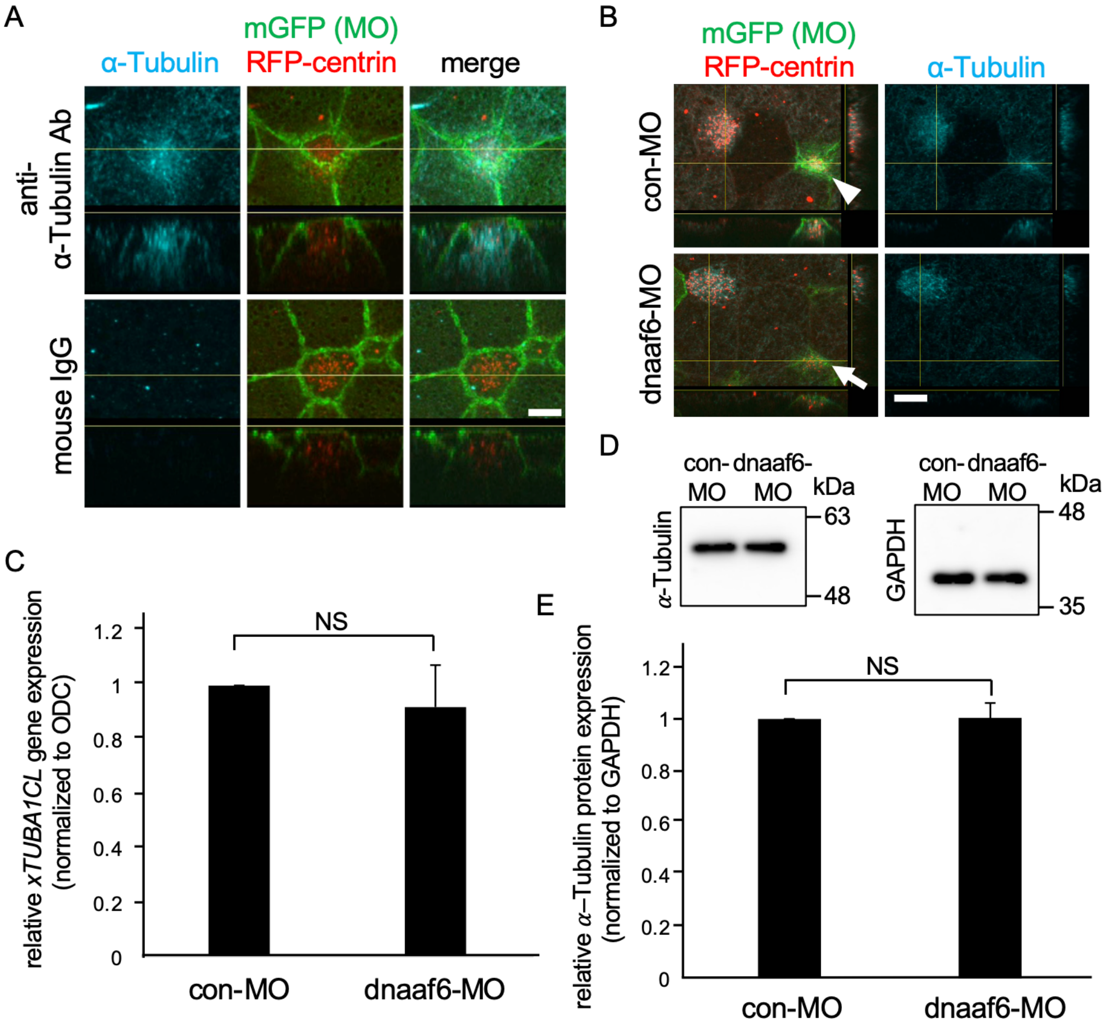

Figure S-6. The amount of *xTUBA1CL* mRNA and α-Tubulin protein were not changed in DNAAF6 depleted *Xenopus* ectodermal explants

A. mouse IgG was used as a negative control for primary antibody of immunohistochemistry. The signal of α-Tubulin protein (cyan) was detected in the samples with anti-α-Tubulin Ab (left upper panel) but not mouse IgG (left lower panel). The scale bar represents 5μm. B. The expression of α-Tubulin protein is abolished in precursor MCCs of *Xenopus* DNAAF6 morphant epidermis at the beginning of ciliogenesis (stage 20 embryos). Fluorescently labeled α-Tubulin protein (cyan) and fluorescently labeled centrin (red), as a maker of basal bodies, were imaged in the cytoplasm of MCCs. One mGFP-positive MCC which received con-MO is indicated by a white arrowhead (left upper panel) and one mGFP-positive MCC which received dnaaf6-MO is indicated by a white arrow (left lower panel). Left panels are the merged images and right panels show only α-Tubulin. The view of x and z plane of the z-stack at the yellow line or the view of y and z plane of the z-stack at the yellow

line in left upper large yellow square was shown under or on the right of left upper yellow square, respectively. The scale bar represents 5μm. C. The expression level of *xTUBA1CL* mRNA in DNAAF6 depleted *Xenopus* ectodermal explants was similar to wild type *Xenopus* ectodermal explants. Three independent experiments were performed. D. There was no difference of α-Tubulin protein expression between wild type *Xenopus* ectodermal explants and DNAAF6 depleted *Xenopus* ectodermal explants. Images were shown from one of three independent experiments. D. Relative expression level of α-Tubulin protein shown in Fig. S-6D.

Date

Data S-1: RNA-seq results to identify mRNAs recognized by LARP6

Movies

Movie S-1A: stage 26 embryo with con-MO

Movie S-1B: Embryo with dnaaf6-MO

Movie S-1C: Embryo with WT-RNA and dnaaf6-MO

Movie S-1D: Embryo with Q171*-RNA and dnaaf6-MO
